# Nonhematopoietic IRAK1 drives arthritis via neutrophil chemoattractants

**DOI:** 10.1172/jci.insight.149825

**Published:** 2022-07-08

**Authors:** Thomas Hoyler, Bettina Bannert, Cédric André, Damian Beck, Thomas Boulay, David Buffet, Nadja Caesar, Thomas Calzascia, Janet Dawson, Diego Kyburz, Robert Hennze, Christine Huppertz, Amanda Littlewood-Evans, Pius Loetscher, Kirsten D. Mertz, Satoru Niwa, Gautier Robert, James S. Rush, Giulia Ruzzante, Sophie Sarret, Thomas Stein, Ismahane Touil, Grazyna Wieczorek, Geraldine Zipfel, Stuart Hawtin, Tobias Junt

**Affiliations:** 1 Department of Autoimmunity Transplantation and Inflammation, Novartis Institutes for BioMedical Research, Basel, Switzerland.; 2 Department of Rheumatology, University Hospital Basel, Basel, Switzerland.; 3 Institute of Pathology, Cantonal Hospital Baselland, Liestal, Switzerland.

**Keywords:** Autoimmunity, Arthritis, Innate immunity

## Abstract

IL-1 receptor-activated kinase 1 (IRAK1) is involved in signal transduction downstream of many TLRs and the IL-1R. Its potential as a drug target for chronic inflammatory diseases is underappreciated. To study its functional role in joint inflammation, we generated a mouse model expressing a functionally inactive IRAK1 (IRAK1 kinase deficient, IRAK1KD), which also displayed reduced IRAK1 protein expression and cell type–specific deficiencies of TLR signaling. The serum transfer model of arthritis revealed a potentially novel role of IRAK1 for disease development and neutrophil chemoattraction exclusively via its activity in nonhematopoietic cells. Consistently, IRAK1KD synovial fibroblasts showed reduced secretion of neutrophil chemoattractant chemokines following stimulation with IL-1β or human synovial fluids from patients with rheumatoid arthritis (RA) and gout. Together with patients with RA showing prominent IRAK1 expression in fibroblasts of the synovial lining, these data suggest that targeting IRAK1 may be therapeutically beneficial. As pharmacological inhibition of IRAK1 kinase activity had only mild effects on synovial fibroblasts from mice and patients with RA, targeted degradation of IRAK1 may be the preferred pharmacologic modality. Collectively, these data position IRAK1 as a central regulator of the IL-1β–dependent local inflammatory milieu of the joints and a potential therapeutic target for inflammatory arthritis.

## Introduction

IL-1 receptor-associated kinase 1 (IRAK1) is a key signaling component of the IL-1R family and all TLRs except TLR3 (1). Ligand binding leads to recruitment of the adaptor molecule myeloid differentiation primary response 88 (MyD88) and subsequent phosphorylation events leading to activation of both IRAK4 and IRAK1 (2). Downstream phosphorylation of IRAK1 initiates signaling through TNF receptor-associated factor 6 and transforming growth factor-β–activated kinase-1 (TAK-1). TAK-1 activates both the mitogen-activated protein kinase and NF-κB pathways for transcription of proinflammatory cytokines (3).

IRAK4 was identified as a key regulator of innate immunity. IRAK4-deficient mice and patients show impaired TLR/IL-1R–dependent immune responses (4, 5). This, together with data showing that IRAK4 kinase-deficient mice are resistant to disease development in models of arthritis (6) and systemic lupus erythematosus (7), provided a rationale for development of IRAK4 antagonists for therapy of autoimmune diseases (8).

Much less is known about the immunological function of IRAK1, but emerging preclinical data suggest that it might become an attractive therapeutic target as well (9). IRAK1-KO mice show some protection in mouse models of colitis and experimental autoimmune encephalitis (10, 11). However, more convincing data support a role of IRAK1 in rheumatological diseases: IRAK1 single nucleotide polymorphisms are linked to an increased risk of rheumatoid arthritis (RA) (12) and particularly systemic lupus erythematosus (SLE) (13–16). In addition, peripheral blood mononuclear cells (PBMCs) from patients with SLE overexpress IRAK1 (17), and mice expressing a catalytically inactive form of IRAK1 are protected in a mouse model of SLE (18). Endosomal TLRs and potentially IL-1R are likely upstream activators of IRAK1 in these diseases; however, in vivo evidence is scarce, and the cellular effector mechanisms that depend on IRAK1 signaling are less clear. For example, genetic inactivation of IRAK1 kinase affects IFN-β cytokine production from mouse plasmacytoid DCs following in vitro stimulation with TLR ligands (19). In contrast, in humans, IRAK1 deficiency has the most profound effect on cytokine production from fibroblasts (20).

In our study, we used a combination of in vivo experimentation and functional studies of patient samples to investigate the role of IRAK1 in innate driven inflammation and more specifically, in joint inflammation. We found that IRAK1 plays a key role in neutrophil recruitment to inflamed joints and identify IRAK1 as a target for inflammatory arthritides. Our results also suggest that degradation of IRAK1, rather than kinase inhibition, is the most suitable mode for intervention.

## Results

### IRAK1 kinase is essential for cell type–specific TLR7 and TLR9 activation in vitro and in vivo.

**Figure 1:**
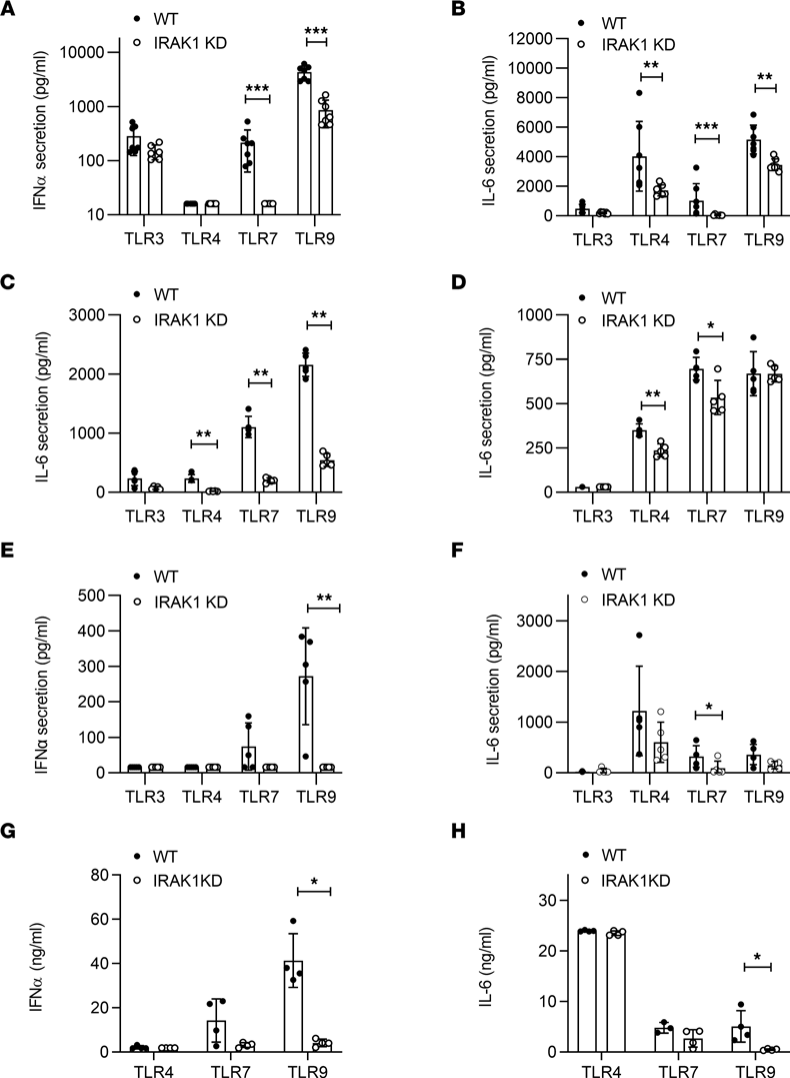
IRAK1KD mice show impaired TLR-mediated cytokine responses. In vitro cytokine release from IRAK1KD and WT cells: (**A**) BMDC/IFN-α, (**B**) BMDC/IL-6, (**C**) B cells/IL-6, (**D**) BMDM/IL-6, (**E**) blood/IFN-α, and (**F**) blood/IL-6 following TLR activation. Plasma levels of (**G**) IFN-α and (**H**) IL-6 following i.v. injection of TLR4, TLR7, or TLR9 agonists in vivo. Data points represent individual mice with means ± SD and are representative of ≥2 (**A**–**F**) and 2 (**G** and **H**) independent experiments, Mann-Whitney *U* test. *P < 0.05, **P < 0.01, ***P < 0.001.

IRAK1 kinase-deficient ([B6-Irak1tm1.1(*K239S)Npa], IRAK1KD) mice were generated by introducing a point mutation (K239S) in the catalytic ATP binding domain (21) that renders IRAK1 nonfunctional (22). Immune cells and fibroblasts from these mice showed normal expression of the IRAK1 and IRAK4 transcripts, yet lower expression of IRAK1 protein (Supplemental Figure 1; supplemental material available online with this article; https://doi.org/10.1172/jci.insight.149825DS1). Reduced IRAK1 protein expression in IRAK1KD mice is consistent with observations in a different IRAK1KD mouse strain harboring the D358A inactivating point mutation (23). In line with the published roles of IRAK1 for TLR signaling, bone marrow dendritic cells (BMDCs) from IRAK1KD mice showed impaired IFN-α production upon stimulation with TLR7 and TLR9 agonists and reduced IL-6 secretion following TLR4, TLR7, and TLR9 stimulation (Figure 1, A and B). Similarly, B cells from IRAK1KD mice showed reduced IL-6 secretion following TLR4, TLR7, and TLR9 stimulation (Figure 1C), while IRAK1KD bone marrow–derived macrophages (BMDMs) showed only weakly impaired TLR responses (Figure 1D). When whole blood was stimulated with TLR4, TLR7, and TLR9 agonists, the response pattern was consistent with the observations in isolated cells: IRAK1KD mice showed reduced IFN-α secretion following TLR7 and TLR9 stimulation and reduced IL-6 secretion following TLR4, TLR7, and TLR9 stimulation, yet the effect did not reach statistical significance for all conditions tested (Figure 1, E and F).

These in vitro results are consistent with published data from IRAK1-KO mice (24) and the kinase-inactive mutant strain IRAK1(D359A) (19, 20, 25). To analyze how the IRAK1(K239S) KD mutation in IRAK1KD mice affected TLR signaling, B cells were purified from WT and IRAK1KD mice and activated with TLR4, TLR7, and TLR9 agonists. As a proximal biomarker, we studied IRAK1 degradation, as this is a necessary step for IRAK1 signaling (26, 27), and as a distal marker, we measured p38 phosphorylation (p-p38) (Supplemental Figure 2A). TLR7 and TLR9 activation, and to a lesser extent TLR4 activation, induced rapid IRAK1 degradation and p-p38 in B cells from WT mice, while total p38 and IRAK4 protein expression levels remained stable (Supplemental Figure 2, B–E). B cells from IRAK1KD mice displayed low IRAK1 protein levels at all times, and IRAK1 degradation and p-p38 signals were barely notable (Supplemental Figure 2A), confirming that IRAK1KD mice expressed a defective IRAK1 mutant. In line with these results, IL-6 release from IRAK1KD B cells was strongly reduced across a range of TLR4, TLR7, and TLR9 agonist concentrations (Supplemental Figure 2F). Following TLR7 and TLR9 stimulation, IL-6 secretion plateaued at lower levels in IRAK1KD B cells compared with WT B cells, likely reflecting the lower IRAK1 expression levels. Together, these data confirm that the K239S mutant in IRAK1KD mice reduced IRAK1 signaling but did not fully abrogate TLR responses.

To appreciate the role of IRAK1 for TLR responses in vivo, we challenged IRAK1KD and WT mice with TLR ligands. Challenge of IRAK1KD mice with TLR7 or TLR9 agonists led to reduced IFN-α (Figure 1G) and IL-6 secretion in plasma (Figure 1H), yet this response only reached statistical significance for TLR9 stimulation. In contrast to our observations in BMDCs, B cells, and BMDMs, TLR4-dependent IL-6 secretion was not impaired in IRAK1KD mice in vivo. Collectively, these results indicate that IRAK1 kinase function regulates TLR-dependent IFN-α and IL-6 production in vitro and in vivo, in a cell type–specific manner.

### IRAK1 affects induction of IFN-stimulated genes and neutrophil recruitment.

**Figure 2:**
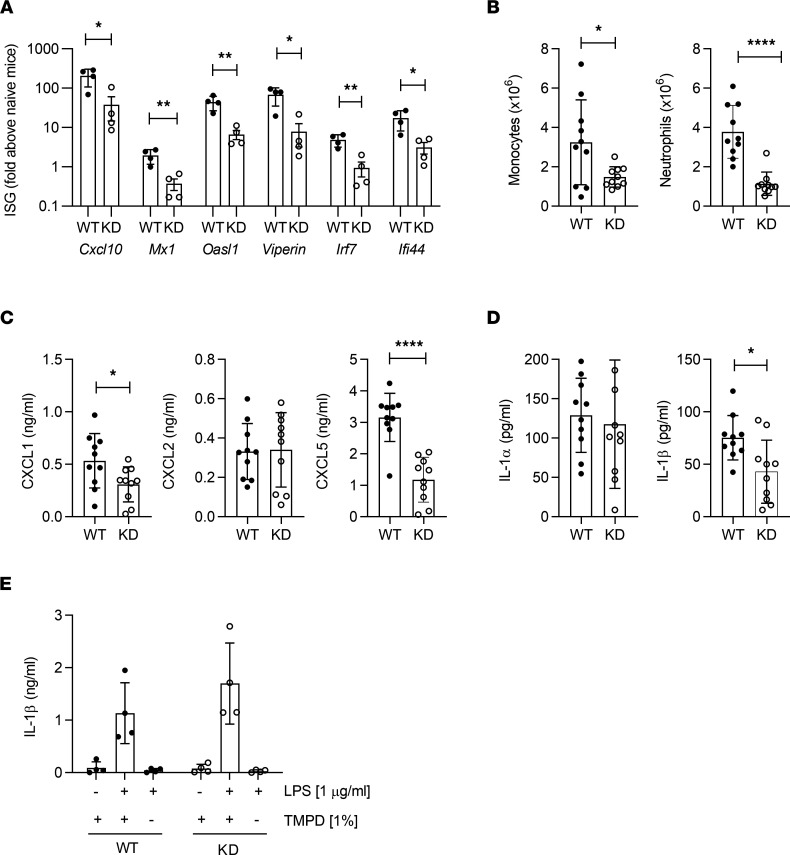
Reduced inflammation of IRAK1KD mice in the TMPD peritonitis model. (**A**) ISG expression by peritoneal exudate cells, 1 representative of 2 independent experiments, means ± SEM, unpaired *t* test. (**B**) Total counts of peritoneal monocytes and neutrophils, (**C** and **D**) CXCL1, CXCL2, CXCL5, IL-1α, and IL-1β in peritoneal lavages. (**B**–**D**) Combined from 2 independent experiments, means ± SD, unpaired *t* test. (**E**) IL-1β secretion following LPS/TMPD stimulation of peritoneal macrophages in vitro. Means ± SD, 1 representative of 2 independent experiments. Two-way ANOVA, Holm-Šidák multiple comparison. Data points in all panels represent individual mice. *P < 0.05, **P < 0.01, ****P < 0.0001.

Based on defective IFN responses in IRAK1KD mice, we investigated the role of IRAK1 in a more complex model of inflammation. The short-term 2,6,10,14-tetramethylpentadecane (TMPD) model shows 2 typical features of rheumatological diseases: expression of IFN-stimulated genes (ISGs) (28, 29) and accumulation of monocytes and neutrophils in inflamed tissues (30, 31). Initially, we analyzed expression of peritoneal ISGs in IRAK1KD and WT mice following TMPD challenge. Consistent with impaired IFN-α responses, IRAK1KD mice showed reduced expression of peritoneal ISGs in the TLR7-dependent TMPD model (32) (Figure 2A). In addition, IRAK1KD mice showed reduced numbers of infiltrating peritoneal monocytes and neutrophils following TMPD injection (Figure 2B). Neutrophil recruitment to the peritoneum in this model critically depends on IL-1R activation by IL-1α, not IL-1β, and on downstream induction of the chemokine CXCL5, and not CXCL1 or CXCL2 (33). TMPD-injected IRAK1KD mice showed normal levels of IL-1α, suggesting that TMPD-induced IL-1α was IRAK1 independent. In contrast, we observed strongly reduced levels of peritoneal CXCL5 (Figure 2, C and D), while there was no reduction in peritoneal CXCL2 in IRAK1KD mice, and CXCL1 was only mildly affected. This allowed us to infer that IRAK1 acts downstream of IL-1α/IL-1R signaling in the TMPD model to induce peritoneal CXCL5 expression and subsequent neutrophil recruitment. The source of peritoneal CXCL5 could have been hematopoietic cells such as adipose tissue macrophages (34) or nonhematopoietic cells, and neutrophils might further potentiate the activity of CXCL5 in the peritoneum via matrix metalloproteinases (35). In contrast to unchanged IL-1α levels, IRAK1KD mice showed a reduction of peritoneal IL-1β following TMPD challenge (Figure 2D). Since IL-1β is an inflammasome product and TMPD is an inflammasome activator (36), we investigated IL-1β secretion from LPS-primed IRAK1KD and WT peritoneal macrophages triggered with TMPD (Figure 2E). Since IRAK1KD macrophages showed normal IL-1β secretion, the reduced peritoneal IL-1β levels within TMPD-challenged IRAK1KD mice were likely not caused by a cell-intrinsic effect on macrophages but rather were due to defective recruitment of IL-1β–expressing myeloid cells.

Altogether, in the TMPD model, IRAK1 plays a critical role for the TLR7-dependent induction of ISGs and for CXCL5-dependent neutrophil recruitment downstream of the IL-1R.

### IRAK1 function drives serum-induced arthritis via neutrophil recruitment.

**Figure 3:**
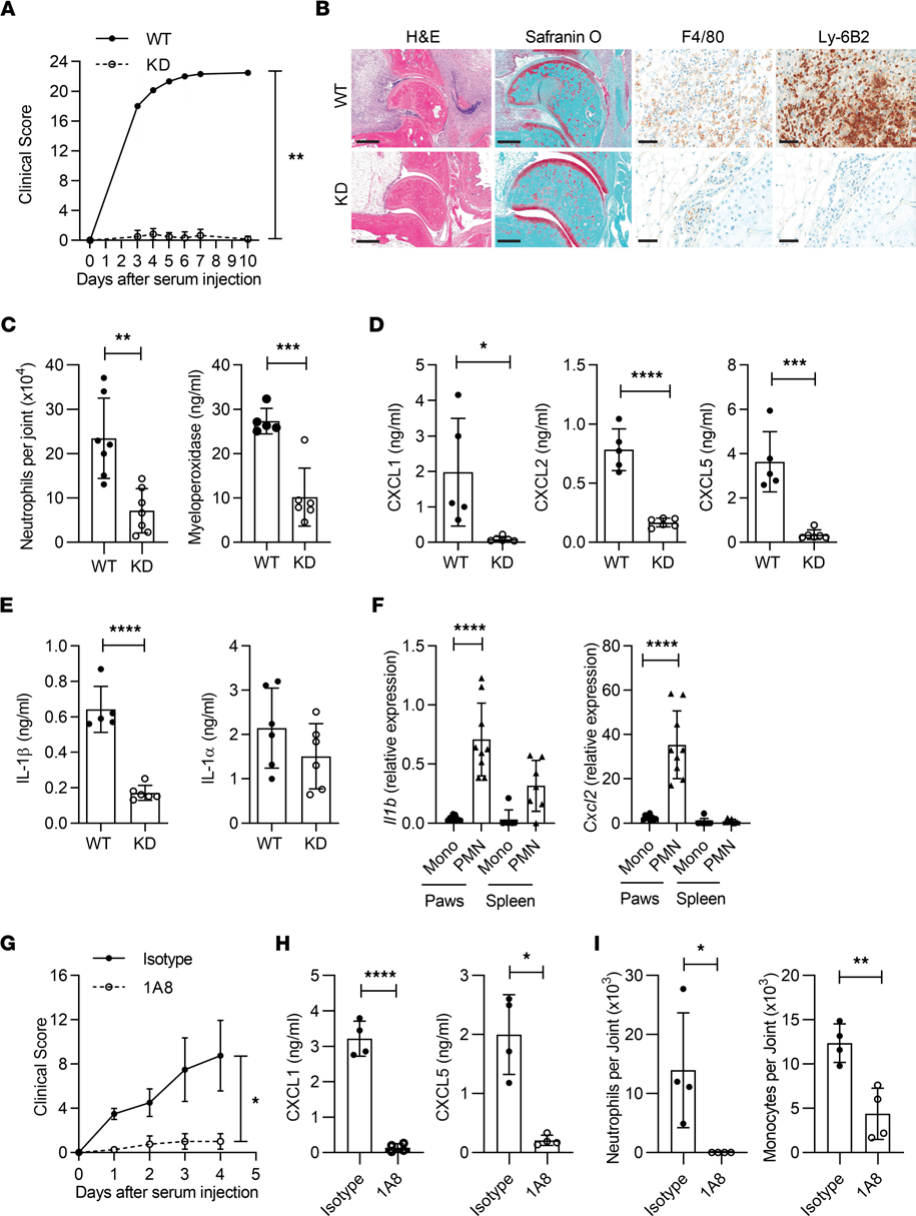
K/BxN arthritis depends on IRAK1-dependent neutrophil recruitment to joints. (**A**) Clinical scores. Data points represent means ± SEM of *n* = 6 mice per group. (**B**) Representative histology of WT and IRAK1KD joints on day 7. Scale bar: H&E, 500 μm; Safranin O, 300 μm; F4/80 and Ly-6B2, 50 μm. (**C**) Neutrophil numbers and MPO expression in ankle joints, means ± SD. (**D** and **E**) Cytokine levels in paw homogenates, means ± SD. Data in **A**–**E** are representative of 2 independent experiments. (**F**) Monocytes (Mono) or neutrophils (PMN) were isolated from inflamed joints or spleens from WT and IRAK1KD animals at day 4 and analyzed for *Il1b* and *Cxcl2* expression, means ± SD. Pooled data from 3 experiments. (**G**) Clinical scores in neutrophil-depleted (1A8) or isotype-treated animals. Data points represent means ± SEM of *n* = 4 mice per group. (**H**) Levels of CXCL1 and CXCL5 in paw homogenates at day 4, means ± SD. (**I**) Neutrophil and monocyte numbers in ankle joints at day 4, means ± SD. Data in **G**–**I** are representative of 2 independent experiments. Data points in **C**–**F**, **H**, and **I** represent individual mice. Paired *t* test (**A** and **G**), unpaired *t* test (**C**–**E**, **H**, **I**), 1-way ANOVA, Bonferroni’s posttest (**F**). *P < 0.05, **P < 0.01, ***P < 0.001, ****P < 0.0001.

With IRAK1’s involvement in IL-1R signaling and subsequent neutrophil recruitment in the TMPD model, we hypothesized that a similar effect may be evident in the IL-1–dependent K/BxN model of serum transfer arthritis (37–39). Surprisingly, joint swelling was completely suppressed in IRAK1KD mice (Figure 3A). In addition, cartilage destruction, as shown by Safranin O staining, as well as macrophage and neutrophil infiltration into joints, were critically dependent on IRAK1 (Figure 3B). Reduced neutrophil numbers in IRAK1KD joints were consistent with reduced local expression of the neutrophil activation marker MPO (Figure 3C) and all tested neutrophil chemoattractants CXCL1, CXCL2, and CXCL5 (Figure 3D).

In the K/BxN model, disease development and elevated levels of IL-1β in joints go in parallel (39). We found that articular expression of IL-1β, but not IL-1α, was IRAK1 dependent (Figure 3E). This suggested that in addition to a direct role on IL-1R signaling, IRAK1 might affect the secretion of IL-1β within joints. To address the role of IRAK1 in IL-1R activation, we injected IL-1β into dorsal air pouches of WT and IRAK1KD mice and found neutrophil recruitment and the local secretion of CXCL1/2/5 to be IRAK1 dependent (Supplemental Figure 3, A and B). This finding suggested that defective IL-1β/IL-1R signaling in IRAK1KD mice may contribute to the reduced local CXCL1/2/5 expression and defective neutrophil recruitment into K/BxN joints. To address how IRAK1 signaling affects articular expression of IL-1β, we sought to identify the IL-1β–producing cell types during the early phase of arthritis. On day 4, only intra-articular neutrophils, and not monocytes, expressed high levels of *Il1b* (Figure 3F). Therefore, in analogy to the TMPD model, we considered it likely that reduced IL-1β production in joints of IRAK1KD mice may be a consequence of reduced local immigration of neutrophils. Neutrophils may thus contribute to their own recruitment in an IL-1β/IL-1R/IRAK1/chemokine–dependent manner. In addition, we found that intra-articular neutrophils expressed CXCL2 (Figure 3F), a chemokine that might further contribute to paracrine neutrophil recruitment (40).

To provide further evidence that neutrophils are key drivers of early joint inflammation, we analyzed K/BxN induced arthritis in mice specifically depleted of neutrophils following treatment with clone 1A8 (41). Here, we observed reduced joint swelling (Figure 3G) and articular expression of CXCL1/5 (Figure 3H). Reduced monocyte infiltration into inflamed joints was also found as a consequence of neutrophil depletion (Figure 3I).

Collectively, our data suggest that in the K/BxN model, neutrophils are central to a vicious cycle of IL-1β production within inflamed joints with IL-1R–IRAK1–CXCL1/2/5–dependent recruitment of additional IL-1β–secreting neutrophils and monocytes, resulting in a local inflammatory infiltrate and disease exacerbation.

### IRAK1 in nonhematopoietic cells is essential for the development of arthritis.

**Figure 4:**
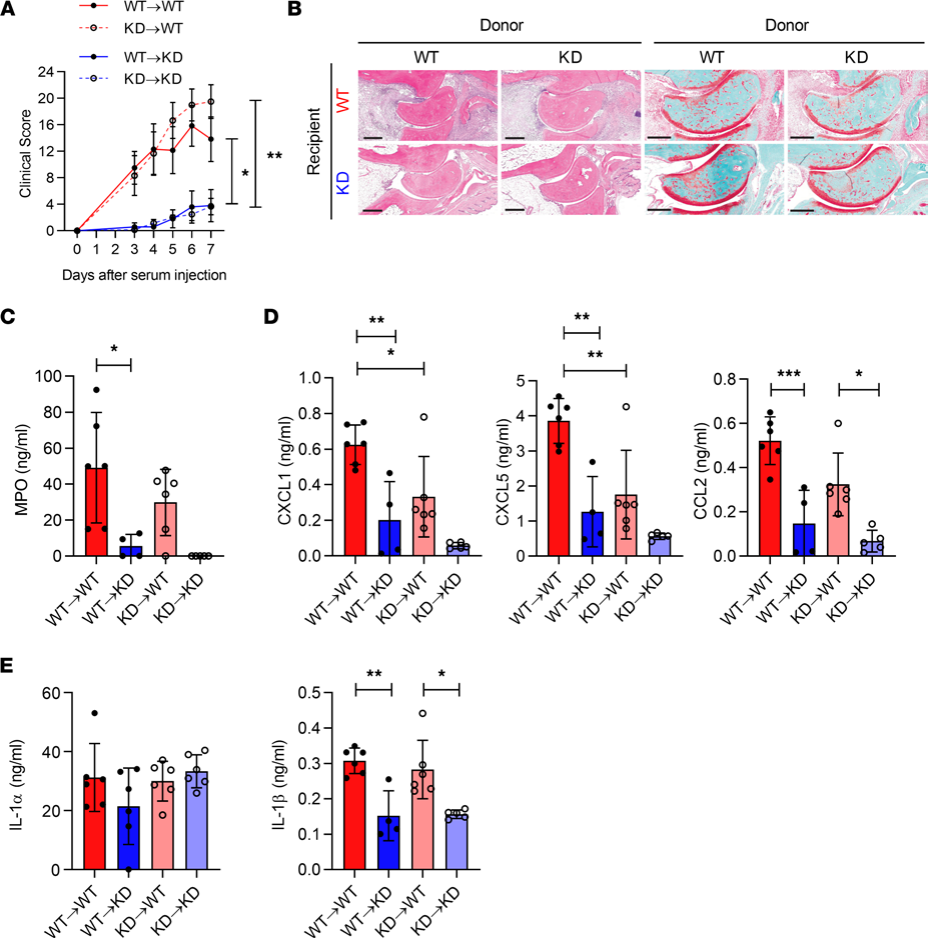
K/BxN arthritis in mixed reciprocal bone marrow chimeras of WT or IRAK1KD mice. (**A**) Clinical index over time; data points represent means of *n* = 6 mice ± SEM. (**B**) Representative H&E staining of joints at day 7. Scale bar H&E, 400 μm; Safranin O, 300 μm. (**C**) MPO, (**D**) CXCL1, CXCL5, CCL2, (**E**) IL-1α, and IL-1β in joints at day 7. Data points in **C**–**E** represent individual mice, means ± SD. One representative of 2 independent experiments. One-way ANOVA with Bonferroni’s posttest. *P < 0.05, **P < 0.01, ***P < 0.001.

Since both hematopoietic and nonhematopoietic cells express IRAK1, we examined which cellular compartment supported IRAK1-dependent arthritis. We generated 4 groups of chimeras of IRAK1KD or WT BM into IRAK1KD or WT recipients and challenged them with K/BxN sera. Our data indicated that only mice expressing WT IRAK1 on radio-resistant cells developed disease and cartilage loss (Figure 4, A and B). Local expression of MPO as a biomarker of neutrophil influx, CXCL1/5, and CCL2 were all reduced in the joints of IRAK1KD recipient mice (Figure 4, C and D). Levels of IL-1α in joints were IRAK1 independent altogether, whereas IL-1β levels were dependent on the presence of IRAK1 in radio-resistant cells (Figure 4E). This again suggested that lower expression of IL-1β in joints of K/BxN mice was not due to a cell-intrinsic effect of IRAK1 in radiosensitive IL-1β–producing cells.

**Figure 5:**
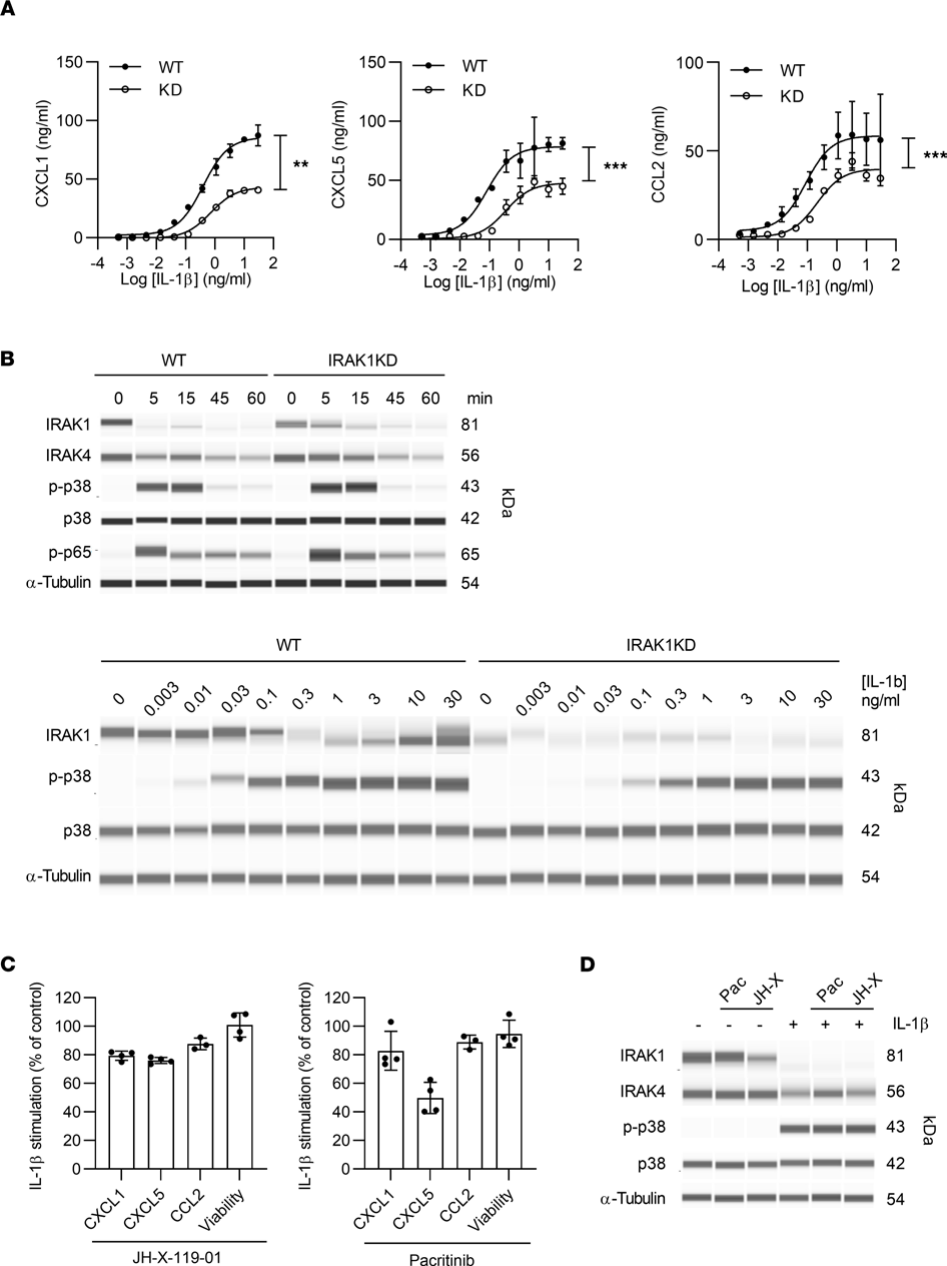
IRAK1 mediates activation of mouse synovial fibroblasts by IL-1β. (**A**) Secretion of CXCL1, CXCL5, and CCL2. (**B**) Protein expression levels. Upper panel, kinetics at 10 ng/mL IL-1β. Lower panel, dose response of IL-1β at 15 minutes. (**C**) CXCL1, CXCL5, and CCL2 secretion and cell viability and (**D**) protein expression of IL-1β–stimulated (10 ng/mL, 15 minutes) WT synovial fibroblasts in presence of IRAK1 inhibitors (1 μM). Data points in **A** and **C** are means ± SD of triplicate measurements, paired *t* test. Data in **A**–**D** are representative of 3, 2, 4, and 2 independent experiments, respectively. **P < 0.01, ***P < 0.001.

In search of the specific radio-resistant cell type that mediated IL-1R/IRAK1–dependent CXCL1/5 production, we initially studied mast cells as they have been proposed as early IL-1β–responsive cells in the K/BxN model (42). However, IL-1β was not a strong driver of either CXCL1 or CXCL5 secretion from bone marrow–derived mast cells (data not shown). Alternatively, synovial fibroblasts are major producers of CXCL1 and CXCL5 in RA (43). Interestingly, we found that IRAK1KD synovial fibroblasts showed significantly reduced CXCL1, CXCL5, or CCL2 secretion across a broad range of IL-1β concentrations (Figure 5A) and IL-1α (data not shown). These data show that IL-1/IL-1R signaling on synovial fibroblasts drives CXCL1/5 and CCL2 secretion in an IRAK1-dependent manner. As observed for TLR stimulation in B cells, IL-1β stimulation of mouse WT fibroblasts led to IRAK1 degradation, p-p38, and phosphorylation of NF-κB p65 (Figure 5B). In IL-1β–stimulated IRAK1KD fibroblasts, IRAK1 degradation was slightly slower, yet p-p38 and p-p65 were observed, consistent with some remaining capacity of IRAK1KD to still respond to IL-1β. When we investigated IRAK1 signaling in WT fibroblasts across a range of IL-1β concentrations, IRAK1 degradation was agonist dependent, and only at high doses a smaller band was observed, possibly the smaller IRAK1b isoform, which is involved in postacute IL-1R signaling (44). p-p38 was notably less sensitive to stimulation in IRAK1KD fibroblasts, but again cells were not completely resistant to IL-1β signaling.

To analyze whether the observed phenotype of impaired cytokine secretion in IRAK1KD fibroblasts was driven by reduced IRAK1 expression levels or kinase deficiency, we used published pharmacological IRAK1 inhibitors with some selectivity over IRAK4. When mouse synovial fibroblasts were stimulated with IL-1β in the presence of the JAK2/Fms-like tyrosine kinase 3/IRAK1 antagonist pacritinib (45) or the covalent IRAK1 antagonist JH-X-119-10 (46), CXCL1/5 and CCL2 were not inhibited, except by a high dose of pacritinib, which may reflect an off-target effect (Figure 5C). Similarly, both compounds did not prevent IRAK1 degradation or p-p38 in fibroblasts (Figure 5D). This suggests that the fibroblast phenotype of IRAK1KD mice is mostly driven by reduced IRAK1 protein expression and not by its impaired kinase function.

### Synovial fluids from patients with RA and gout elicit CXCL1 and CXCL5 from synovial fibroblasts via IRAK1.

**Figure 6:**
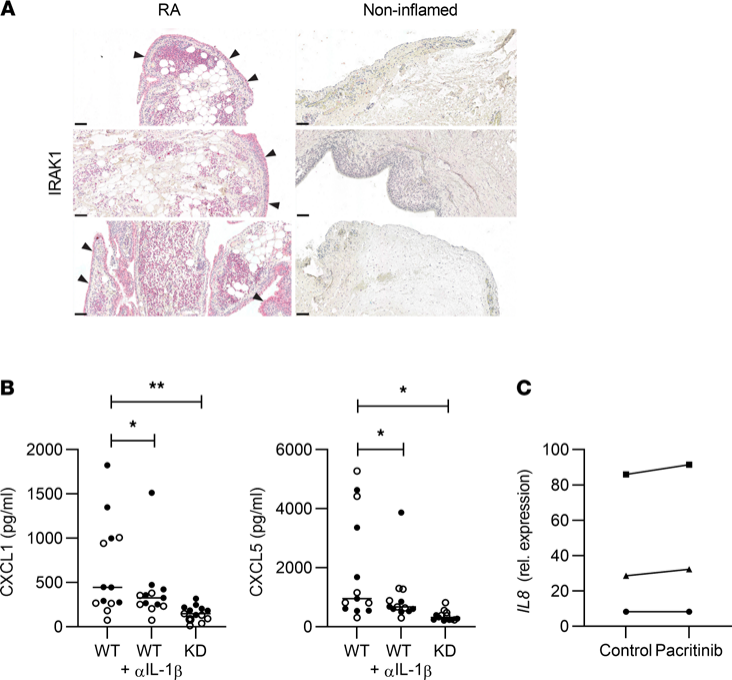
IRAK1 is essential for induction of neutrophil chemoattractants by RA synovial fluids and IL-1β. (**A**) IRAK1 expression in joints from RA patients and noninflamed controls; arrowheads indicate IRAK1 in synovial membranes; scale bar: 100 μm. (**B**) CXCL1 and CXCL5 stimulated by synovial fluids from patients with RA (*n* = 7, closed symbols) or gout (*n* = 6, open symbols) from murine WT, anti–IL-1β–treated (10 mg/mL), or IRAK1KD synovial fibroblasts. Data points represent individual patients, means of triplicates or quadruplicate measurements, horizontal lines: medians, 1-way ANOVA, Dunnett’s posttest. (**C**) Expression of *IL8* in human RA synovial fibroblasts stimulated with 3 different human RA synovial fluids in the absence or presence of pacritinib (1 μM). Data points are means of triplicate measurements. *P < 0.05, **P < 0.01.

To translate the above findings to humans, joint sections of patients with RA were stained for IRAK1 (Figure 6A). Consistent with our functional results in mice, IRAK1 was detected not only in infiltrating immune cells but also in fibroblasts of the thickened synovial membrane. As observed for mouse synovial fibroblasts, synovial fibroblasts from patients with RA released the neutrophil chemoattractant IL-8 and induced p-p38 following IL-1β stimulation (Supplemental Figure 4A). For other TLR-driven responses on human synovial fibroblasts, only TLR3 activation was evident as described (47). As TLR3 does not signal via MyD88/IRAK1, this was not examined further. Similar to the mouse model results, pharmacological inhibition of IRAK1 did not strongly prevent cytokine secretion or p-p38, except with high doses of the poly-kinase inhibitor pacritinib (Supplemental Figure 4B). For both mouse and human RA fibroblasts, inhibition of IRAK1 kinase activity did not appear sufficient to abrogate IL-1β–induced signaling and cytokine release.

Activation of synovial fibroblasts by synovial fluid is a key driver of the local proinflammatory milieu in patients with RA (48–50). Based on our findings in mice, we hypothesized that synovial fluid from patients with RA or gout can activate synovial fibroblasts in an IL-1R/IRAK1–dependent manner. The levels of IL-1β and IL-8 in synovial fluid of patients with RA correlated well (Supplemental Figure 5A), which further encouraged us to explore a functional relationship.

To address if synovial fluid from patients with RA could activate the IL-1R/IRAK1 pathway in synovial fibroblasts, we preselected patients with IL-1β in their synovial fluids. Since we were unable to obtain IRAK1-deficient human fibroblasts, and since human IL-1β activates murine IL-1R (51), we used synovial cells from WT and IRAK1KD mice to investigate the role of IRAK1 for release of neutrophil chemoattractants. Indeed, synovial fluid from multiple patients with RA or gout elicited CXCL1 and CXCL5 release from murine synovial fibroblasts (Figure 6B). Pretreatment of synovial fluids with a neutralizing antibody against human IL-1β significantly reduced CXCL1 and CXCL5 by an average of 25% and 32%, respectively (*n* = 13, Figure 6B). However, the effects using IRAK1KD fibroblasts were always more profound than IL-1β–dependent effects alone (average of 72% and 66% reduction of CXCL1/5 secretion, respectively). This suggested that other IRAK1-dependent stimuli within synovial fluids might contribute as well. While the mean reduction by anti–IL-1β was comparatively small, it is interesting to note that a small subset of RA and gout synovial fluids showed stronger decreases of CXCL1/5 secretion upon anti–IL-1β treatment (Supplemental Figure 5B). Taken together, these data show that synovial fluids from patients can elicit chemokines from mouse synovial fibroblasts via IRAK1. However, this outcome could be a result of lower IRAK1 protein expression or defective IRAK1 kinase function. To distinguish the two, we treated human RA synovial fibroblasts with human RA synovial fluids in the presence of pacritinib and assessed IL-8 mRNA levels, as the synovial fluids contained substantial amounts of IL-8. Pacritinib did not reduce RA fluid–induced IL-8 transcription in RA synovial fibroblasts (Figure 6C). Together with our previous results, this indicated that reduced IRAK1 expression in IRAK1KD fibroblasts was most likely responsible for the observed in vitro results and the in vivo phenotype in the K/BxN model. With that, our data suggest that inhibition of IRAK1 kinase function is likely not sufficient for arthritis treatment, and that development of a drug that degrades IRAK1 protein may be of more merit. In summary, our study positions IRAK1 as an attractive pharmacological target for joint inflammation. Antagonism of the IL-1β/IRAK1/neutrophil axis, preferably by degrading IRAK1 protein, has the potential to break a vicious circle of disease amplification in inflamed joints.

## Discussion

**Figure 7:**
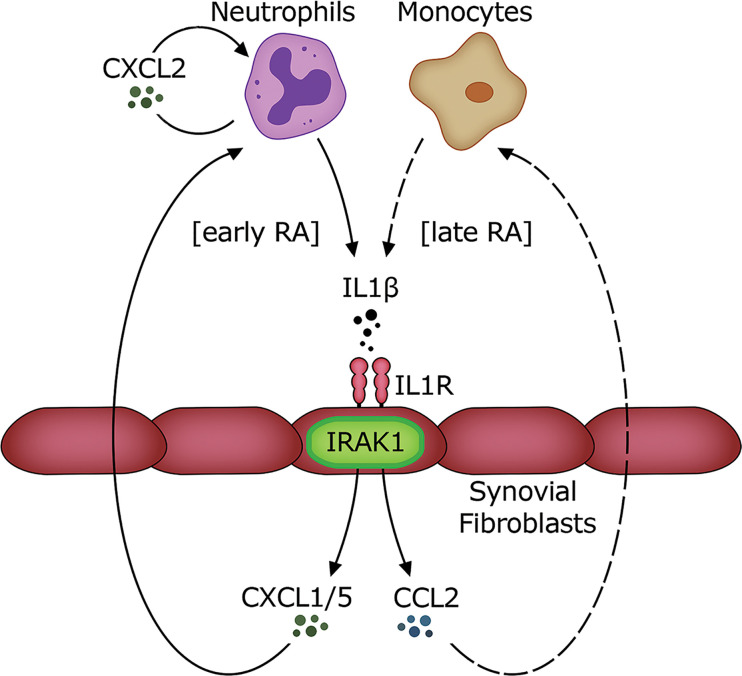
Proposed model of the role of IRAK1 in joint inflammation. Neutrophils are recruited to affected joints early in disease and secrete IL-1β. This activates synovial fibroblasts and potentially other nonhematopoietic cells to secrete neutrophil chemoattractants CXCL1 and CXCL5 and the monocyte chemoattractant CCL2 via IRAK1. Paracrine CXCL2 may further amplify neutrophil accumulation within joints. The resulting amplification of neutrophil and monocyte influx leads to disease exacerbation in inflamed joints.

In this study we defined a cell- and context-specific role for IRAK1 kinase activity in murine models of autoinflammation. IRAK1 not only regulated expression of type I IFNs and ISGs downstream of TLR7/9 signaling but more importantly played a key role on synovial fibroblasts for IL-1R–driven secretion of neutrophil chemoattractants. This was confirmed in translational studies using human RA synovial fibroblasts and synovial fluids from patients with RA and gout. The data presented in this study allow us to propose a model of IRAK1 involvement in joint inflammation (Figure 7): neutrophils producing IL-1β are recruited to inflamed joints early in disease, possibly by an IL-1α–mediated effect. Elevated levels of IL-1β lead to IL-1R/IRAK1–dependent activation of synovial fibroblasts to secrete CXCL1/5 and recruit more neutrophils. This leads to a perpetual cycle of neutrophil influx, likely enhanced by paracrine CXCL2 secretion from neutrophils. Synovial fibroblasts activated by IL-1β/IL-1R/IRAK1 also secrete the monocyte chemoattractant CCL2. This together with locally recruited monocytes may further enhance local IL-1β release in the joints and exacerbate inflammation. Collectively, IL-1R/IRAK1–dependent signaling on synovial fibroblasts is central to the accumulation of a monocyte/neutrophil-rich infiltrate in joints.

Our systematic study using a potentially novel IRAK1KD mouse strain also revealed fundamental knowledge about IRAK1. First, expression of a functionally deficient IRAK1 form in mice reduced IRAK1 protein but not transcript levels. This is biologically relevant, as it was also observed in another IRAK1KD strain (23), and it suggests that catalytic activity of IRAK1 regulates its own protein half-life. This is conceivable as IRAK1 phosphorylation and degradation are functionally connected (27). It remains to be seen how chronic pharmacological IRAK1 kinase inhibition affects protein stability. Second, the IRAK1(K239S) mutation still facilitates TLR and IL-1β signaling, albeit at lower agonist sensitivity and magnitude. Finally, IL-1R signal strength appears to be a defining parameter of the outcome of IRAK1 signaling in fibroblasts: strong activation of IL-1R leads to appearance of a shorter IRAK1 isoform, possibly to limit overstimulation of cells (44), while weaker signals entail IRAK1 degradation. With that, our results are in line with other authors (26) showing that IRAK1 kinase activity sensitizes IRAK1 for degradation.

Since IRAK signaling is at the convergence point of several well-validated targets for inflammatory diseases such as TLR7/8/9 and IL-1R signaling, IRAK4 antagonists (8) have advanced to clinical trials for multiple autoimmune indications. We considered IRAK1 as an equally attractive target directly downstream of IRAK4. Furthermore, given that its mechanistic role is less well defined, we considered it important to study its proinflammatory function in vitro and in vivo. Our in vivo studies were particularly revealing because the strong in vivo effect of IRAK1 was in stark contrast to the relatively milder effects on immune cells in vitro.

Preclinical evidence on the involvement of IRAK1 in autoimmune diseases has emerged over the past years, but many studies had limitations. For example, one of the strongest genetic predictors for SLE development is the Xq28 risk locus, encompassing Irak1 and methyl CpG binding protein 2 (Mecp2) (13, 52). A recent study in mice proposed *Irak1* as the key risk gene within this locus (17), but it could not functionally discriminate the pathogenic roles of IRAK1 and IRAK4, because it relied on an IRAK1/4 dual antagonist. Similarly, IRAK1-KO mice develop a milder form of lupus-like disease (15), but this IRAK1 targeting strategy led to complete disruption of the IRAK1/4 signalosome, thus not allowing inferences on the potential effect of only selectively targeting IRAK1. To address these shortcomings, the functionally deficient IRAK1(D358A) strain was generated and revealed reduced ISG expression following TLR signaling in vitro (19). A cross of this mutation into the ABIN1(D485N) lupus model suggested that IRAK1 catalytic function affects disease progression independent of the type I IFN pathway (18). The translational value of these data is unclear, particularly as elevated ISG expression is a hallmark of human lupus (53).

Here we extend these studies with a different IRAK1KD strain and show that functional IRAK1 deficiency impairs TLR7/9 signaling and IFN-I pathway activity in vivo. More importantly, we uncovered an unexpected role of IRAK1 in the development of joint inflammation in mice. IRAK1 was key for neutrophil recruitment by synovial fibroblasts via IL-1R signaling. However, pharmacological inhibition of IRAK1 in these systems with currently available kinase inhibitors did not replicate these findings. This provides preclinical evidence for IRAK1 as a potential target for joint inflammation yet suggests that targeting IRAK1 protein degradation may be a preferred therapeutic approach.

Of note, IRAK4 kinase activity in nonhematopoietic cells was proposed to be essential for RA development (6). Other studies pointed to an important role of neutrophil recruitment in K/BxN serum transfer arthritis (37, 39, 54–57). Here we were able to connect the IRAK pathway to neutrophil recruitment by demonstrating IRAK1-dependent secretion of neutrophil chemoattractants from stromal cells in the K/BxN model. Finally, we could extend these observations from mice to humans with functional studies of patient samples.

In arthropathies, fibroblast-like synoviocytes (FLSs) have recently gained attention since they are (a) in close contact with bone structure and disease-causing osteoblasts in the intimate lining of the joints, (b) activated by infiltrating neutrophils, and (c) able to secrete diverse cytokines for leukocyte recruitment and activation (58). Thus, there is increasing evidence for the stromal milieu as a key regulator of arthritis development (59). We found that synovial fibroblasts were strong producers of neutrophil chemoattractants such as CXCL1 and CXCL5 and monocyte chemoattractant CCL2. Release of these important chemokines was dependent on IRAK1 kinase activity. Our results using mouse bone marrow chimeras do not fully exclude that other radio-resistant cells such as synovial macrophages contribute to the observed effect. Nonetheless, our data allow the conclusion that IRAK1 is essential for shaping the local milieu within inflamed joints, and they support the notion that FLSs contribute to disease through crosstalk with leukocytes (60). Our studies using human synovial fluids confirmed that IL-1β was a key trigger for neutrophil chemoattractant release from FLSs. In line with a previous report (33), we found that IL-1α can play a similar role and direct CXCL5 release, at least in the TMPD model.

What is the primary source of synovial IL-1β in joint inflammation? Published data in the K/BxN model and our findings indicate a role for immune complex–activated neutrophils (61). Human neutrophils have been shown to secrete IL-1β following activation by immune complexes as well (62). Since human synovial fibroblasts are sensitive to IL-1β, it is likely that neutrophils contribute to their own recruitment via an IL-1R/IRAK1–driven feed-forward loop. However, we cannot exclude monocytes as other key contributors to this IL-1R/IRAK1–dependent feed-forward loop.

The above described biological function of IRAK1 kinase activity on FLSs is for the most part congruent with data from patients with a large genomic deletion of *IRAK1* (and *MECP2*). PBMCs from these patients respond normally to IL-1 and all TLR stimuli, whereas patient fibroblasts are functionally impaired downstream of all TLRs and to some extent IL-1R (20).

Interference with IL-1R signaling shows limited clinical efficacy in RA (63–65), and we found that some patients have detectable levels of IL-1β in synovial fluids. However, our analyses of synovial fibroblasts suggest that inhibition of the IRAK1 pathway could offer advantages beyond IL-1R inhibition, as synovial fluids may contain endogenous TLR agonists in addition to IL-1β. Finally, our evidence for a role of IRAK1 on synovial fibroblasts and emerging data on an IL-1β/neutrophil axis in patients with inflammatory bowel disease (66) suggest that fibroblasts within other organs may show a similar phenotype. This may extend the range of target indications for future IRAK1-based therapies.

This study defines a specific in vivo role of IRAK1 function in and beyond TLR signaling. The most outstanding finding was the nonredundant role of IRAK1 for production of neutrophil and monocyte chemoattractants by synovial fibroblasts downstream of the IL-1R. This mechanism defines the local immune infiltrate in the TMPD model and the K/BxN model of autoinflammation. Our translational data using RA patient biopsies, synovial fibroblasts, and synovial fluids from patients with RA and gout show that our results may extend to human disease as well. With that, IRAK1 may have potential as a therapeutic target in human arthropathies.

## Methods

### TLR ligands.

The TLR agonists PolyIC, CpG type B (ODN1826), and CpG type A (ODN1585) were purchased from Invivogen; R848 from Enzo; and LPS from MilliporeSigma. ssRNA40 (5′-GCCCGUCUGUUGUGUGACUC-3′, phosphothioate-linked) and R0006 (5′-UUGUUGUUGUUGUUGUUGUU-3′, phosphothioate-linked) were custom synthesized by MicroSynth. Dioleoyl-3-trimethylammonium propane (DOTAP) was used for complexation of TLR7 and TLR9 ligands ssRNA40 and ODN1585, respectively (Roche).

### Mice.

Wild-type C57BL/6J mice were from Charles River. IRAK1KD [B6-Irak1tm1.1(*K239S)Npa] mice were generated as follows: murine IRAK1 genomic sequences containing the kinase-inactive mutation (K239S) were synthesized and integrated into a targeting vector. Mouse C57BL/6 ES cells were transfected by electroporation and homologous recombination confirmed in neomycin-selected clones. A clone with verified sequences in the recombination region was injected into Balb/C host blastocysts and transferred into pseudo-pregnant CB6F1 foster mothers. Germline transmission was confirmed in the offspring of male chimeric mice, which were bred to C57BL/6 Cre deleter females (Taconic) to remove the neo cassette. As IRAK1 is located on the X chromosome, female offspring were heterozygous. IRAK1-modified mice were crossed with C57BL/6 mice to remove the Cre recombinase gene. IRAK1KD mice showed no signs of susceptibility to infections, no developmental defects, and normal cellular distribution of major immune subsets in spleen, bone marrow, thymus, and mesenteric lymph nodes (Supplemental Table 1). All mice were used in sex-matched groups, at 6–14 weeks of age.

### TLR stimulation of mouse BMDCs.

To generate BMDCs, pooled bone marrow cells from individual IRAK1KD or WT mice were resuspended in RPMI1640 with GlutaMAX (supplemented with 10% FBS, penicillin/streptomycin, 2-mercaptoethanol at 0.05 mM, Thermo Fisher Scientific) and cultured in the presence of 100 ng/mL Flt3L (PeproTech, BioConcept Switzerland) for 7–10 days. Expansion of CD11b^–^B220^+^CD11c^+^PDCA1^+^ plasmacytoid DCs from WT and IRAK1KD bone marrow was not different based on FACS analysis (antibodies, Supplemental Table 2) using an LSRFortessa (BD Biosciences) and FlowJo v.7.6.5 (TreeStar). BMDCs (60,000 cells/well) were stimulated with TLR agonists as indicated in Supplemental Table 3. IFN-α (ELISA, Invitrogen) and IL-6 (HTRF, Cisbio) in supernatants were quantified according to each manufacturer’s protocol.

### Isolation and stimulation of mouse splenic B cells.

B cells were isolated from spleens of individual mice using the EasySep mouse B cell isolation kit (STEMCELL Technologies). B cell purity was more than 90% as determined by FACS using CD19 and CD45R/B220 (Supplemental Table 2). Isolated B cells (200,000 cells/well) were stimulated with TLR agonists as indicated in Supplemental Table 3. IL-6 was quantified by DuoSet ELISA (eBioscience) using a SpectraMax plate reader with SoftmaxPro V5 software (Molecular Devices).

For cell signaling studies, pooled B cells (2 × 10^6^ cells) were stimulated with TLR agonists at times indicated in the figures prior to lysis in RIPA buffer (MilliporeSigma) containing protease and phosphatase inhibitors (MilliporeSigma). Protein expression of IRAK1, IRAK4, NF-κB p65, p-p38, and total p38 was analyzed by automated capillary-based immunoassay system (WES) using standard protocols (ProteinSimple). In brief, each lysate was diluted with 5× Fluorescent Master Mix (ProteinSimple) and incubated with each primary antibody together with appropriate loading control as indicated in Supplemental Table 2. Samples were loaded onto a 12–230 kDa WES Separation Module 25 capillary cartridge and quantification of each protein was determined using the Compass software v6.0.0 (ProteinSimple).

### TLR stimulation of mouse BMDMs.

To generate BMDMs, bone marrow cells pooled from individual mice were cultured for 7 days in RPMI1640 GlutaMAX-supplemented media as above for BMDCs, including M-CSF (40 ng/mL, R&D Systems). BMDMs (100,000 cells/well) were stimulated with TLR agonists as indicated in Supplemental Table 3. Quantification of IL-6 was determined by ELISA as above.

### TLR stimulation of mouse blood.

Mouse blood was collected into citrate tubes, diluted 1:1 in RPMI1640, and stimulated with agonists for TLR3 (30 μg/mL PolyIC precomplexed with 25 μg/mL DOTAP), TLR4 (LPS, *E. coli* 0111:B4, 1 μg/mL), TLR7 (1 μg/mL ssRNA40 precomplexed with 25 μg/mL DOTAP), or TLR9 (0.2 μM or 1 μM ODN1585 precomplexed with 25 μg/mL DOTAP for IFN-α and IL-6, respectively). The concentration of each TLR agonist was selected as >EC_80_ of the maximal response, based on previously determined responses on WT blood. After 20 hours at 37°C, IFN-α (ELISA) and IL-6 (HTRF) were quantified in supernatants.

### Expression of IRAK1 in specific cell types.

For protein expression of IRAK1, splenocytes, B cells, BMDMs (2 × 10^5^/mouse), and fibroblasts (3 × 10^4^/mouse) from 5 mice were pooled and lysed in RIPA buffer containing protease and phosphatase inhibitors (MilliporeSigma). Protein expression of IRAK1 and controls was analyzed by automated capillary-based immunoassay system (WES) as described above. For mRNA expression of IRAK1 and IRAK4, RNA was extracted from indicated cell types of individual mice using the RNeasy Mini Kit (QIAGEN) and blood using QIAamp RNA blood mini kit (QIAGEN). Isolated RNA was reverse-transcribed using the Reverse Transcription with high-capacity cDNA kit (Applied Biosystems). Expression of IRAK1 and IRAK4 was determined using TaqMan Gene Expression Master Mix (Applied Biosystems), TaqMan probes (Supplemental Table 4), and a ViiA 7 Real-Time PCR System (Applied Biosystems) using the 2-ΔΔCt method with fold change = 2-Δ(ΔCt), where ΔCt = Cttarget – CtHPRT control and Δ(ΔCt) = ΔCtsample – ΔCtcontrol sample.

### In vivo challenge with TLR agonists.

Mice were injected intravenously with agonists for TLR7 (ssRNA0006, 20 μg/mouse complexed with DOTAP), TLR9 (ODN1585; 20 μg/mouse complexed with DOTAP), or TLR4 (LPS *E. coli* *0127:B8*; 5 mg/kg) for 2 hours. IFN-α (Invitrogen) and IL-6 (R&D Systems) in plasma were quantified by ELISA.

### TMPD peritonitis model and in vitro TMPD challenge.

Female IRAK1KD mice and WT controls (*n* = 5 per group) were injected i.p. with 0.5 mL TMPD (MilliporeSigma). On day 7, peritoneal cavities were lavaged using ice-cold HBSS with 2% *v/v* FBS, then centrifuged (300*g*, 10 minutes, 4°C), and CXCL1, CXCL2, CXCL5, CCL2, IL-1α, and IL-1β were quantified from supernatants by ELISA (R&D Systems). Total cell counts of peritoneal lavages were determined, and neutrophils (CD3^-^CD19^–^CD11b^+^Ly6G^+^Ly6C^+^) and inflammatory monocytes (CD3^–^CD19^–^CD11b^+^Ly6G^–^Ly6C^+^) were analyzed by FACS after Fc receptor blockade (anti-CD16/CD32, Supplemental Table 2). ISGs were quantified from peritoneal cells by quantitative PCR as indicated above, using TaqMan probes as indicated in Supplemental Table 4. Means of triplicates were calculated and fold induction of each transcript was calculated from individual mice by comparing with naive mice.

Thioglycollate-induced peritoneal macrophages were elicited by i.p. injection of 0.5 mL 3% thioglycollate (MilliporeSigma) and collected on day 4 in ice-cold PBS supplemented with 1 mM EDTA and 2% heat-inactivated FBS). Cells were primed for 4 hours with LPS (1 μg/mL, *E. coli* 0111:B4) prior to stimulation with 1% (w/v) TMPD (MilliporeSigma) for 16 hours. IL-1α and IL-1β were determined from supernatants by ELISA as above.

### Dorsal air pouch model.

Subcutaneous air pouches were formed on the back of mice by injection of 5 mL sterile air with injection repeated on day 3. IL-1β (500 μg/mL in saline) was injected into pouches on day 6. After 6 hours, exudate was extracted using ice-cold PBS with 2% *v/v* FBS and centrifuged at 350*g* for 5 minutes at 4°C. Levels of CXCL1, CXCL2, CXCL5, and CCL2 were quantified from supernatants by ELISA. Peritoneal exudate cells were counted, and neutrophils (CD11b^+^Ly6G^+^Ly6C^+^) and inflammatory monocytes (CD11b^+^Ly6G^–^Ly6C^+^) were analyzed by FACS after Fc receptor blockade (anti-CD16/CD32, Supplemental Table 2).

### K/BxN serum transfer model of arthritis.

Arthritis was induced in IRAK1KD and control mice by intravenous injection of 150 μL pooled serum from K/BxN arthritic mice. Severity of paw swelling was determined by scoring within metatarsal and ankle regions (ranging 0–3) for each paw with a maximum clinical index of 24 per mouse. Scoring criteria: 0 = no detectable sign of inflammation; 1 = slight edema in the paw; 2 = more pronounced swollen paw; 3 = ankylosis or severely swollen paw. For histology, hind paws were fixed in 10% (*v/v*) buffered formalin for 48 hours, decalcified for 6 days in Immunocal (Decal Chemical Corp), and embedded in paraffin. Sections were stained with H&E or Safranin O. Neutrophils were visualized using anti–Ly-6B2 and rabbit anti-rat polyclonal secondary antibody (antibodies, Supplemental Table 2) and macrophages using anti-F4/80 and Histofine SimpleStain Mouse MAX PO (Nichirei Biosciences Inc), on a Ventana Discovery XT with the DABMap detection kit (Roche). Homogenates from paw tissue were prepared by removing the skin of the tissue before transferring the joints into 0.5 mL RIPA lysis buffer (Thermo Fisher Scientific) plus cOmplete Protease Inhibitor (Roche) in tissue homogenizing CKmix tubes (Precellys). Samples were homogenized using a Precellys 24 tissue homogenizer (twice at 5500 rpm, 20 seconds). Levels of CXCL1, CXCL2, CXCL5, MPO (R&D Systems), IL-1α, and IL-1β from paw homogenates were quantified by ELISA.

Ankle joints were extracted from skin and treated with DNase I (0.1 mg/mL, Roche) plus collagenase IV (0.5 mg/mL, Gibco) in PBS for 30 minutes at 37°C with agitation. Splenocytes from the same animals were isolated and resuspended in PBS supplemented with 2% FCS and 2-mercaptoethanol (0.1 mM). Neutrophils were identified on a BD LSRFortessa as CD45^+^CD3^–^CD19^–^CD11c^–^CD11b^+^MHCII^–^Ly6C^int^Ly6G^+^ and monocytes as CD45^+^CD3^–^CD19^–^CD11c^–^CD11b^+^MHCII^–^Ly6C^hi^Ly6G^–^ (antibodies, Supplemental Table 2). Cells were counted by FACS acquisition for a fixed time and flow rate, or by adding 123count eBeads (eBioscience). For gene expression analysis, neutrophils and monocytes from paws and spleens were sorted on a FACSARIA III (BD Biosciences) under medium pressure with an 80 μm nozzle to avoid activation of cells, into RNAlater (Thermo Fisher Scientific). RNA was extracted and reverse-transcribed using the high-capacity cDNA kit (Applied Biosystems). Gene expression analysis was performed using PowerUp SYBRGreen PCR master mix (Thermo Fisher Scientific) with primers as indicated (Supplemental Table 4), and expression values were calculated relative to β2-microglobulin (b2M) and GAPDH using the 2-ΔΔCt method with fold change = 2-Δ(ΔCt), where ΔCt = Cttarget – ([Ctb2M + CtGAPDH]/2). In vivo depletion of neutrophils was achieved by injection of 25 mg/kg 1A8 (Supplemental Table 2) at 48 hours prior to study, with rat IgG2a isotype (BioXCell) as a control. For generation of BM chimeras, 8-week-old mice were irradiated twice (2 × 4.5 Gy), prior to i.v. injection of 5 × 10^6^ bone marrow donor cells per animal; monitored for engraftment via a CD45.1 congenic markers; and used up to 10 or 12 weeks following bone marrow transplantation (≥90% chimerism).

### Immunohistochemistry of human knee joint samples.

FFPE knee biopsies from 3 RA patients and 3 noninflamed controls (Supplemental Table 5) were sectioned (5 μm), treated with Bond Epitope Retrieval Solution for 20 minutes, and incubated with anti-IRAK1 (sc-5288, Santa Cruz Biotechnology) at 1:50 for 30 minutes. Stainings were developed using the Bond Polymer Refine Red Detection kit (Leica Biosystems).

### Ex vivo stimulation of murine and human synovial fibroblasts.

Ankle joints from WT or IRAK1KD mice were dissected, incubated at 37°C in DMEM containing 0.4 mg/mL collagenase IV and 10% FBS for 45 minutes, vortexed, and filtered over a 70 μm cell strainer (Falcon, Corning). Fibroblasts were resuspended in DMEM supplemented with 10% (*v/v*) FBS (Gibco), 100 U/mL penicillin, and 100 μg/mL streptomycin. Cells (<5 passages) were stimulated with murine IL-1β (PeproTech) in the absence or presence of JH-X-119-01 (MedChem Express) and pacritinib (in-house synthesis). After 16 hours at 37°C, CXCL1, CXCL5, and CCL2 were quantified from supernatants by ELISA (R&D Systems). Alternatively, after incubation at 37°C for the indicated times, protein expression was quantified from lysates by automated capillary-based immunoassay system (WES) as indicated above. Stimulation of synovial fibroblasts with patient synovial fluid (10% in culture medium) was performed in the absence or presence of 10 μg/mL anti-human IL-1β antibody (canakinumab) (67). Human RA synovial fibroblasts were stimulated with 3 μg/mL PolyIC, 100 ng/mL LPS, or 0.1 ng/mL human IL-1β in the absence or presence of JH-X-119-01 or pacritinib. Protein expression was quantified from lysates at the indicated time points by an automated capillary-based immunoassay system and secreted IL-8 measured by HTRF.

### In vitro differentiation and stimulation of mast cells.

Bone marrow cells extracted from tibia and femurs of mice were pooled following filtration across a 40 μm screen and red blood cell lysis (BioConcept). Bone marrow–derived mast cells (5 × 10^5^ cells/mL) were differentiated with 5 ng/mL murine IL-3 (PeproTech) in IMDM with l-glutamine (Gibco) supplemented with 10% FBS, penicillin/streptomycin, sodium pyruvate (1 mM), 2-mercaptoethanol (0.2 mM), HEPES (25 mM), and 1× nonessential amino acids (Thermo Fisher Scientific). Cells were supplemented with fresh growth medium and harvested after 3 weeks, resulting in more than 70% immature FceR1^+^Kit^+^ mast cell precursors as assessed by FACS. Cells were stimulated with various TLR ligands or with different concentrations of IL-1β as indicated and incubated for 16 hours at 37°C before quantifying levels of CXCL1 or CXCL5 as indicated above.

### Statistics.

Data were analyzed using Excel XL fit 5.0 (Microsoft) with XLfit add-in (IDBS; version 5.2.0) or using Prism v.7.03 (GraphPad Software) using nonlinear regression for ELISA standard curves. Bar graphs show individual mice and means of groups ± SD unless indicated otherwise. Statistical significance between groups was calculated using a 2-tailed paired or unpaired *t* test for comparison of 2 groups, and ordinary 1-way or 2-way ANOVA with the indicated posttest, using GraphPad Prism. *P < 0.05, **P < 0.01, ***P < 0.001, ****P < 0.0001 for all tests. *P* < 0.05 was considered statistically significant.

### Study approval.

All animal studies were performed in accordance with Swiss Federal laws, and all experimental procedures were reviewed and approved by the Cantonal Veterinary Office of Basel-Stadt (Basel, Switzerland). Synovial fluids from patients with RA were obtained from Asterand Biosciences. Synovial fluids from patients with gout were received from the Department of Rheumatology, University Hospital Basel, Switzerland. Synoviocytes from a patient with RA were obtained from Articular Engineering (Northbrook, Illinois, USA). Studies were conducted in accordance with ethical principles originating in the 1975 Declaration of Helsinki and approved by the local Ethics Committee (Ethikkommission Nordwest- und Zentralschweiz project 2021-01445 and 2016-01324) (Basel, Switzerland). All participants provided written informed consent prior to the study and were never identifiable by name.

## Author contributions

TH, GW, CH, TC, JD, PL, ALE, SN, SH, and TJ designed the study. BB and DK provided human biosamples. TH, CA, G Robert, D Beck, TM, KDM, D Buffet, NC, G Ruzzante, SS, RH, IT, TS, TB, and GZ conducted experiments and evaluated results. TH, TC, ALE, PL, JSR, GW, SH, and TJ contributed to manuscript writing. SH and TJ agreed about TJ being last and corresponding author as TJ mentored TH and monitored in vivo studies, while SH provided background knowledge and monitored most in vitro studies.

## Supplementary Material

Supplemental data

## Version 1 07/08/2022

Electronic publication
