# Supplementary material for: Nonhematopoietic IRAK1 drives arthritis via neutrophil chemoattractants
Source: JCI Insight. 2022 Jul 8;7(13):e149825. doi: 10.1172/jci.insight.149825 (PMC9310529; doi:10.1172/jci.insight.149825)
Supplement: Supplemental data [file jciinsight-7-149825-s008.pdf]

Supplemental Data

Supplemental Figures

Supplemental Figure 1: Expression of IRAK1 across multiple cell types from WT and IRAK1KD mice

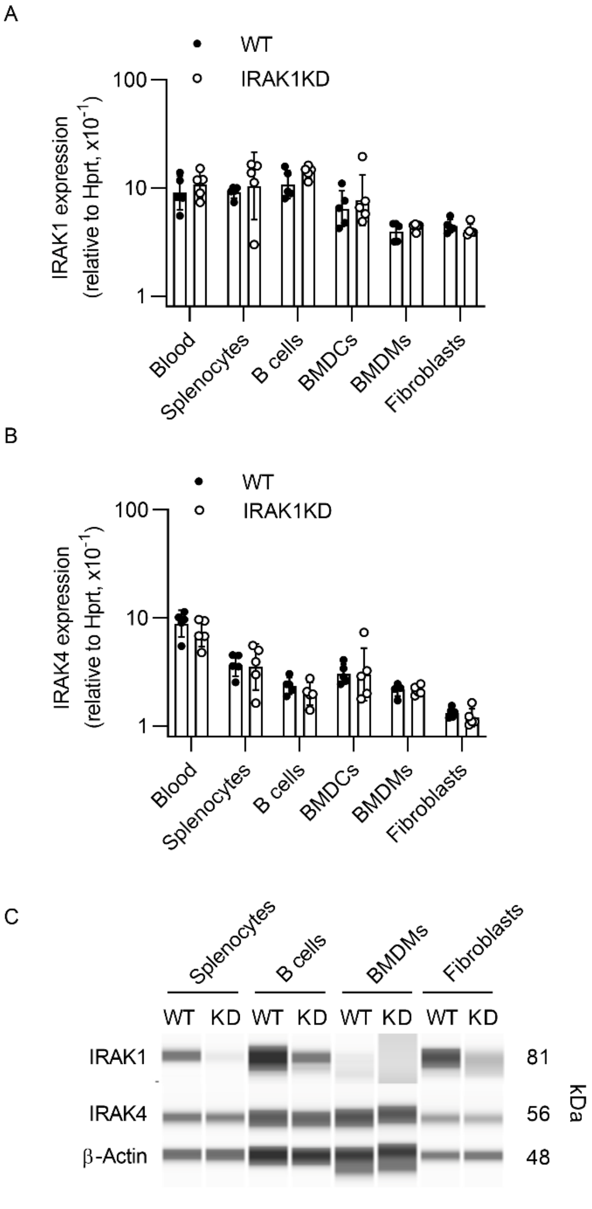

Expression of (**A**) IRAK1 and (**B**) IRAK4 transcript in indicated cell types. Data points are individual mice, geomeans ± SD, unpaired t-test. (**C**) IRAK1 and IRAK4 protein expression in different cell types. Data are from five pooled mice per lane.

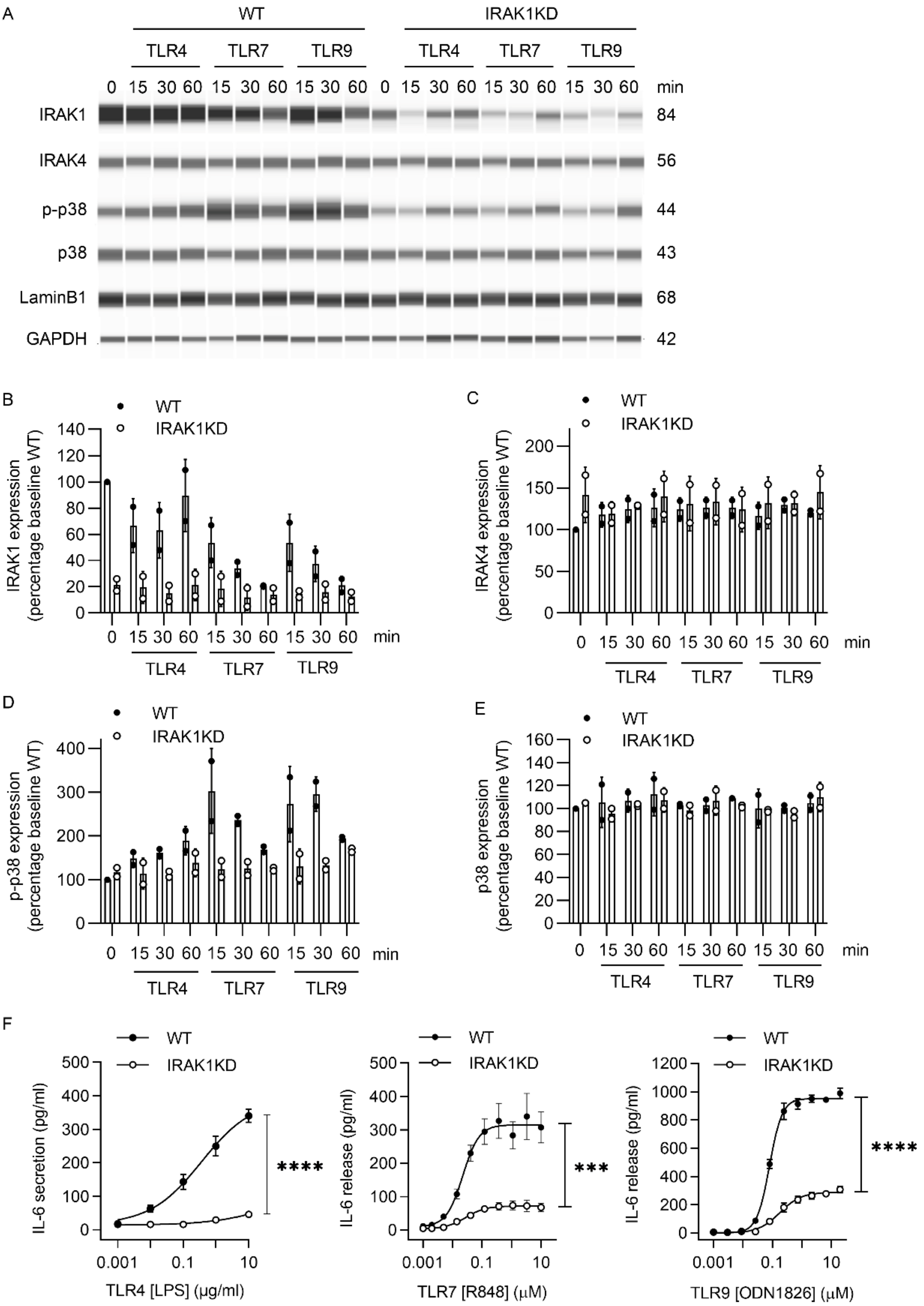

(**A**) Protein expression in B cells from WT and IRAK1KD following stimulation with TLR ligands (TLR4: 1 mg/ml LPS; TLR7: 1 mM R848; TLR9: 1 mM ODN1826). Expression of (**B**) IRAK1, (**C**) IRAK4, (**D**) p-p38 and (**E**) p38, normalised to protein loading control and relative to WT baseline. Data are representative (**A**) or means ± SD (**B-E**) of two independent experiments, B cells were pooled from five

WT and IRAK1KD mice. (**F**) IL-6 secretion upon TLR4, TLR7 and TLR9 stimulation. Data points represent means of five individual mice with ± SD; one representative of 2-3 independent experiments, unpaired t-test.

Supplemental Figure 3: Reduced neutrophil recruitment in IRAK1KD mice after *in vivo* IL-1β challenge

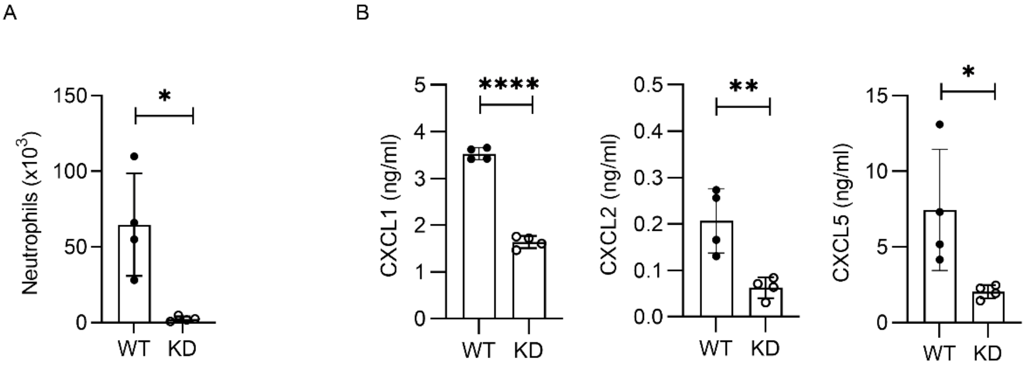

(**A**) Total counts of infiltrating neutrophils and (**B**) Chemokine secretion in air pouch exudates 6 h after injection of IL-1β. Data points are individual mice, means ± SD. One representative of two independent experiments, unpaired t-test.

Supplemental Figure 4: Responses of human RA synovial fibroblasts to TLR and IL-1β stimulation

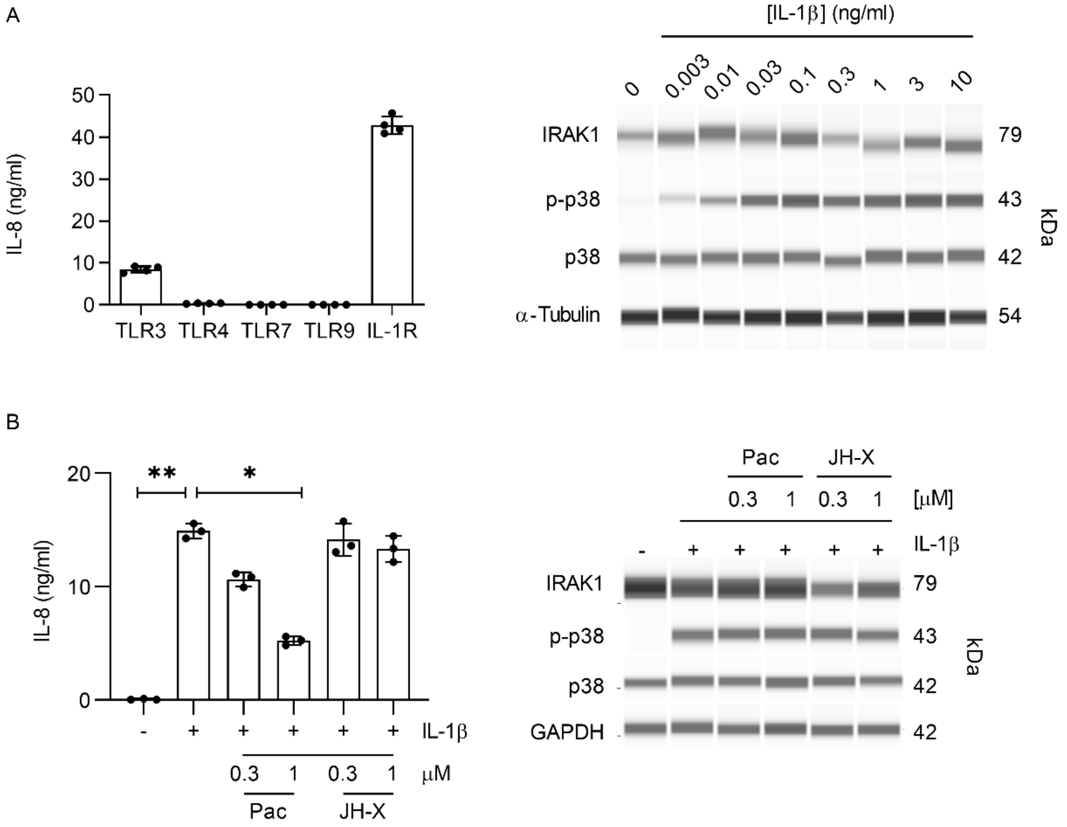

(A) Left, IL-8 secretion, bars represent means of quadruplicate measurements ± SD. Right, protein expression by capillary Western Blot from human RA synovial fibroblasts following IL-1β stimulation. One representative of two independent experiments. **(B)** Left, IL-8 secretion upon IL-1β stimulation (0.1 ng/ml for 15 min, i.e. EC80) and IRAK1 inhibitor treatment, bars represent means of triplicate measurements ± SD, Kruskal-Wallis test with Dunn's post-test. Right, protein expression by capillary Western Blot from human RA synovial fibroblasts. One representative of two independent experiments.

Supplemental Figure 5: **Analysis of human synovial fluids**

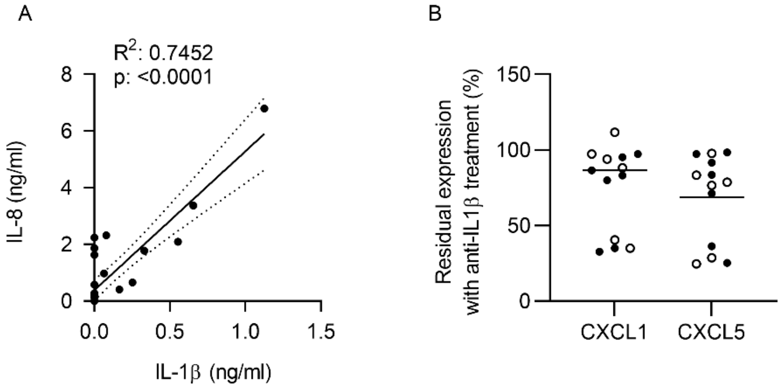

(**A**) IL-8 and IL-1β protein in individual human RA synovial fluids. Pearson correlation, dotted lines represent 95% confidence interval, (**B**) Residual expression of CXCL1 or CXCL5 from WT synovial fibroblasts treated with RA (n=7 patients, filled symbols) or gout (n=6 patients, open symbols) synovial fluids in presence of anti-IL1β, compared to untreated WT synovial fibroblasts; data from Figure 6B. Horizontal bars, medians.

Supplemental Tables

Supplemental Table 1: Immune cell counts of IRAK1KD and WT mice

**Thymus**		**Total**	**CD4-CD8-**	**CD4+CD8+**	**CD4+**	**CD8+**
	**WT**	79.4 ± 20.6	3.9 ± 1.9	59.8 ± 8.6	9.2 ± 5.7	2.3 ± 1.2
	**IRAK1KD**	95.8 ± 27.6	7.8 ± 5.2	65.4 ± 18.6	16.6 ± 7.6	4.4 ± 2.5
**Spleen**		**Total**	**CD4$^+$**	**CD8$^+$**	**CD4$^+$CD8$^-$ CD25$^+$Foxp3$^+$**	**CD3$^+$γδTCR$^+$**
	**WT**	54.4 ± 10	11.3 ± 3.6	8.0 ± 2.0	1.3 ± 0.3	0.3 ± 0.1
	**IRAK1KD**	46.3 ± 18.3	9.1 ± 3.9	6.7 ± 2.6	1.1 ± 0.5	0.3 ± 0.1
		**CD11b$^+$ Ly6G/C^{low}**	**CD11b$^+$ Ly6G/C^{dim}**	**CD11b$^+$ Ly6G/C^{high}**	**CD11c^{dim}CD11b$^-$B220$^+$ pDC**	**CD11c^{high}CD11b$^-$B220$^-$ cDC**
	**WT**	2.0 ± 0.5	0.8 ± 0.1	1.0 ± 0.5	0.7 ± 0.1	0.2 ± 0.1
	**IRAK1KD**	1.9 ± 0.6	0.7 ± 0.3	0.7 ± 0.3	0.7 ± 0.3	0.3 ± 0.1
**MLN**		**Total**	**CD4$^+$**	**CD8$^+$**	**CD19$^+$**	**CD11c^{dim}CD11b$^-$ B220$^+$ pDC**
	**WT**	12.8 ± 6.0	5.8 ± 3.9	3.8 ± 1.6	4.1 ± 1.9	0.1 ± 0.1
	**IRAK1KD**	15.0 ± 4.7	6.8 ± 2.2	4.4 ± 1.2	4.6 ± 1.6	0.1 ± 0.04
**BM**		**Total**	**B220$^+$CD43$^+$ CD19$^+$ proB**	**B220$^+$CD43$^-$ IgM$^-$preB**	**B220$^+$CD43$^-$ IgM$^+$ Immature B**	**B220^{high}CD43 IgM$^+$ mature B**
	**WT**	24.2 ± 13.8	0.6 ± 0.2	2.9 ± 0.8	1.0 ± 0.4	2.1 ± 0.6
	**IRAK1KD**	22.2 ± 4.4	0.4 ± 0.08	2.1 ± 0.7	0.8 ± 0.4	1.3 ± 0.6

Counts [x10^6] of cell subsets in different compartments as evaluated by flow cytometry. MLN, mesenteric lymph nodes, BM, bone marrow. Means ± SD of 8 mice.

Supplemental Table 2: Antibodies

**Flow cytometry**		
**Antigen**	**Clone**	**Company**
CD3	145-2c11	eBioscience
CD4	RM4-5	BD Biosciences
CD8a	53-6.7	eBioscience
CD11b	M1/70	eBioscience
CD11c	HL3	BD Biosciences
CD16/32	2.4G2	eBioscience
CD19	1D3	BD Biosciences
CD21/35	7E9	Biolegend
CD23	B3B4	Biolegend
CD25	PC61.5/BC96	eBioscience
CD43	S7	BD Biosciences
CD45	30-F11	Biolegend
CD45R / B220	RA3-6B2	Biolegend
c-Kit	2B8	eBioscience
F4/80	BM8	eBioscience
FcεR1	MAR-1	eBioscience
FoxP3	FJK-16S	eBioscience
GR-1 / Ly6G/C	Rb6-8C5	BD Biosciences
GL-7	GL-7	Biolegend
IgM	11/41	eBioscience
Ly6C	HK1.4	Biolegend
Ly6G	1A8	Biolegend
PDCA-1	927	Biolegend
TCRγδ	UC7-13D5	eBioscience
F4/80	Cl:A3-1	AbD Serotec
**Immunohistochemistry**		
Ly-6B2	7/4	AbD Serotec
F4/80	Cl:A3-1 AbD	AbD Serotec
Rabbit-anti-rat IgG	polyclonal	Vector Laboratories
**Protein detection (WES)**		
Anti-IRAK1	D51G7	Cell Signaling
Anti-pIRAK4 (Thr345/Ser346)	D6D7	Cell Signaling
Anti-IRAK4	2H9	Abcam
Anti-GAPDH	D16H11	Cell Signaling
Anti-p38 MAPK	#9212	Cell Signaling
Anti-Phospho-p38 (Thr180/Tyr182)	#9211	Cell Signaling
Anti-Phospho-NF-κB p65 (Ser536)	93H1	Cell Signaling
Anti-Lamin B1	AF8525	R&D Systems
Anti-alpha Tubulin	11H10	Cell Signaling
Anti-β Actin	AC-15	Cell Signaling
Anti-rabbit-HRP detection module for WES	DM-001	ProteinSimple
Anti-mouse-HRP detection module for WES	DM-002	ProteinSimple

All monoclonal antibodies are directed against mouse antigens unless indicated otherwise.

Supplemental Table 3: Conditions for cytokine release from WT and IRAK1KD cells.

	TLR ligand concentrations for cytokine release					
	**TLR3** (PolyIC*)	**TLR4** (LPS)	**TLR7** (R848)	**TLR7** (ssRNA*)	**TLR9** (ODN1585)	**TLR9** (ODN1826)
**BMDCs (IFNα)**						
WT (EC_{50})	10 ± 0.47 (2)	>10,000 (2)	-	0.69 ± 0.63 (13)	160 ± 81 (6)	-
IRAK1KD (EC_{50})	10 ± 0.31 (3)	>10,000 (3)	-	>30 (14)	>10,000 (6)	-
For Fig. 1	30	1000	-	1	1000	
**BMDCs (IL-6)**						
WT (EC_{50})	5.5 ± 6.1 (2)	120 ± 130 (2)	-	0.57 ± 0.26 (14)	-	3.8 ± 5.6 (5)
IRAK1KD (EC_{50})	10.6 ± 0.77 (3)	100 ± 70 (3)	-	>30 (10)	-	4.0 ± 6.5 (7)
For Fig. 1	30	1000	-	3	-	100
**B cells (IL-6)**						
WT (EC_{50})	>30 (2)	390 ± 330 (10)	21 ± 5 (6)	-	-	105 ± 37 (10)
IRAK1KD (EC_{50})	>30 (2)	>10,000 (10)	27 ± 16 (6)	-	-	306 ± 175 (10)
For Fig. 1	30	1000	1000	-	-	1000
**BMDMs (IL-6)**						
WT (EC_{50})	>30 (7)	0.55 ± 0.27 (8)	-	0.45 ± 0.20 (6)	-	15 ± 4 (7)
For Fig. 1	30	10	-	1	-	100

Specific cells were isolated as indicated in Materials and Methods and stimulated with TLR agonists: TLR3 (PolyIC*, μg/ml), TLR4 (LPS, ng/ml), TLR7 (R848, nM), TLR7 (ssRNA*, μg/ml), TLR9 (ODN1585, nM) and TLR9 (ODN1826, nM). ODN1585 (i.e. CpG-A) was only used for elicitation of IFNα. The optimal TLR7 agonist for robust IFNα secretion was ssRNA/DOTAP and used for all BMDC experiments. Since B cells respond only weakly to ssRNA/DOTAP, R848 was instead used. Effective potency (EC_{50}) values are geomeans ± SD of (n) separate experiments with each dataset performed in duplicates. The concentrations of TLR ligands for each cell type used in Figure 1 were selected based on an approximate EC_{90} for the respective WT cell type or using a concentration that gave the maximum stimulated response. *: presence of DOTAP (25 μg/ml).

Supplemental Table 4: qPCR Primer sequences

**Taqman primers and probes**	Cxcl10	Mm99999072_m1
	Mx1	Mm01218004_m1
	Oasl1	Mm00455082_m1
	Rasd2	Mm00491265_m1
	Irf7	Mm00516791_g1
	Ifi44	Mm00505670_m1
	Ifi44l	Mm00518988_m1
	Hprt	Mm00446968_m1
	IRAK1	Mm00434254_m1
	IRAK4	Mm00459443_m1
**Primers for SYBRgreen**	Cxcl1	Fwd: 5’-GTG TTG CCC TCA GGG CC-3’
		Rev: 5’-GCC TCG CGA CCA TTC TTG-3’
	Cxcl2	Fwd: 5’-ACG CCC CCA GGA CCC-3’
		Rev: 5’-CTT TTT GAC CGC CCT TGA GA-3’
	Cxcl5	Fwd: 5’-CTC GCC ATT CAT GCG GAT-3’
		Rev: 5’-CTT CAG CTA GAT GCT GCG GC-3’
	Ccl2	Fwd: 5’-TGG CTC AGC CAG ATG CAG T-3’
		Rev: 5’-TTG GGA TCA TCT TGC TGG TG-3’
	IL1b	Fwd: 5’-TGG CAA CTG TTC CTG-3’
		Rev: 5’-GGA AGC AGC CCT TCA TCT TT-3’
	b2m	Fwd: 5’-CCG AAC ATA CTG AAC TGC TAC GTA A-3’
		Rev: 5’-CCC GTT CTT AGC ATT TGG A-3’

Supplemental Table 5: Characteristics of patients for knee joint immunohistochemistry

Patient	Sex	Age	Description
RA 1	M	65	Thickening of synovial layer, inflammatory infiltrate
RA 2	M	71	Hyperplastic synovial membranes, infiltrations. Krenn synovitis score 7
RA 3	F	58	Hyperplastic synovial membrane, infiltrated. Krenn synovitis score 9
Non-inflamed 1	F	55	Single-layered synovial membrane, no infiltrate
Non-inflamed 2	M	71	Fibrotic connective tissue, no inflammation
Non-inflamed 3	M	73	Fibrotic scar tissue with histiocytic reaction
